# Novelty, diversity, and genetic dark matter in enterococci of invertebrates

**DOI:** 10.1128/mbio.01234-26

**Published:** 2026-08-20

**Authors:** Bruna F. Sgardioli, Matthew C. Phillips, Arjun Miklos, Kailey Fleiszig-Evans, Abigail L. Manson, Suelen Scarpa de Mello, Terrance Shea, Aysun Urhan, Ryan Whipple, Rauf Salamzade, Jasper Sanders, Paulo A. V. Borges, Maria de Lurdes Nunes Enes Dapkevicius, Ashlee M. Earl, Michael S. Gilmore

**Affiliations:** 1 Faculty of Agricultural and Environmental Sciences, University of the Azores 513104 , Angra do Heroísmo, Portugal; 2 Institute of Agricultural and Environmental Research and Technology (IITAA), University of the Azores 513104 , Angra do Heroísmo, Portugal; 3 Division of Infectious Diseases, Department of Medicine, Massachusetts General Hospital 2348, Boston, Massachusetts, USA; 4 Harvard Medical School 1811 , Boston, Massachusetts, USA; 5 Infectious Disease and Microbiome Program, Broad Institute 33577, Cambridge, Massachusetts, USA; 6 Department of Ophthalmology, Mass Eye and Ear, Harvard Medical School 1811 , Boston, Massachusetts, USA; 7 Delft Bioinformatics Lab, Department of Intelligent Systems, Delft University of Technology 2860, Delft, Netherlands; 8 Department of Medical Microbiology and Immunology, School of Medicine and Public Health, University of Wisconsin—Madison 53678, Madison, Wisconsin, USA; 9 University of Azores, CE3C—Centre for Ecology, Evolution and Environmental Changes, Azorean Biodiversity Group, CHANGE—Global Change and Sustainability Institute, School of Agricultural and Environmental Sciences, Angra do Heroísmo, Azores, Portugal; Massachusetts Institute of Technology, Cambridge, Massachusetts, USA

**Keywords:** *Enterococcus*, host association, invertebrate microbiome, vertebrate microbiome, antibiotic resistance, genomics, evolutionary biology, microbial ecology

## Abstract

Enterococci appear to have originated in the guts of early terrestrializing arthropods and invertebrates over 425 million years ago—hosts that are now highly diverse and widespread in nature today. Yet most knowledge of the genus comes from human infection-associated lineages with genomes swollen by the recent accretion of foreign DNA conveyed by mobile elements. Because invertebrates dominate terrestrial animal diversity and biomass, they would be predicted to constitute a major but little-explored reservoir of enterococcal diversity. We therefore systematically examined *Enterococcus* association and species diversification in invertebrate hosts of the comparatively natural, isolated, but well-characterized environment of the Azorean island of Terceira. Over 100 invertebrate specimens were examined for associated enterococci, which were taxonomically classified by whole-genome sequencing. Supporting the existence of a large pool of uncharacterized enterococci and *Enterococcus*-adapted genes, 40% (eight of 20) of the *Enterococcus* species identified were either undescribed, including four candidate new species described here, or very recently discovered. In contrast, control isolates from vertebrates were exclusively of known species typical of sampling elsewhere, discounting geographic isolation as a main driver of the novelty observed. Further, because of the abundance of *E. casseliflavus* and *E. flavescens* in this collection, we obtained the resolution necessary to quantify the divergence and decipher the drivers of speciation in the controversial division between these naturally vancomycin-resistant species. These findings provide robust support for the existence of a large pool of new species and unexplored adaptive traits in invertebrate-associated enterococci—diverse environmental survival traits optimized for expression in an enterococcal background, and well positioned for transmission into human-associated enterococcal strains.

**IMPORTANCE:**

Enterococci are auxotrophic gut-associated bacteria that co-evolved with their terrestrial hosts over many eons. In the last 75 years—the “antibiotic era”*—E. faecalis* and *E. faecium* gained genes for antibiotic resistance and enhanced virulence, emerging as leading causes of multidrug-resistant infection. Little is known about the source of those genes or the pathway by which they entered human-associated strains. A recent global survey suggested a potentially large repository of uncharacterized genetic diversity in the enterococci of invertebrates. We directly tested this prospect by examining enterococci of invertebrate hosts in a largely natural and pastoral environment. Our findings provide clear evidence that invertebrates naturally harbor vast unexplored enterococcal diversity. Moreover, associations are likely driven by intrinsic host selection factors rather than geographic isolation. This expands our knowledge of *Enterococcus* biodiversity, including the identification of four novel species, identifying a vast reservoir of enterococcal genes available to species that colonize and infect humans.

## INTRODUCTION

Much of what we currently know about bacteria of the genus *Enterococcus* stems from the emergence of two species, *E. faecalis* and *E. faecium*, as leading causes of multidrug-resistant human infection. However, in nature, enterococci are ubiquitous inhabitants of the guts of nearly all land animals, and neither *E. faecalis* nor *E. faecium* is exclusive to humans (1). The enterococci appear to have emerged in parallel with the movement of animals onto land—first invertebrates including arthropods about 425 million years ago, and then 50–100 million years later, vertebrates (2). All known *Enterococcus* species are auxotrophic for both vitamins and amino acids, nutritional conditions met in the guts of most animals. It is believed that the necessity of host-to-host transmission, with periods of exposure to an arid environment in between, selected for the stress tolerance genes and traits that distinguish enterococci from more marine-associated microbes, the vagococci, with which they share common ancestry (2). As a result, enterococci likely proliferated, adapted, and diversified first in the guts of land arthropods as the animals themselves evolved, and then began moving up the food chain as vertebrates terrestrialized tens of millions of years later. As arthropods still constitute by far the largest and most diverse terrestrial animal biomass and occur in nearly every ecological niche, they likely retain the largest and most diverse biomass of enterococci. Indeed, the ubiquity of enterococci in insects has been known since pioneering studies in the 1970s (3).

A recent global survey examined *Enterococcus* species associations with various types of hosts (4). Most specimens from vertebrate hosts, which constituted the majority of samples analyzed, yielded known species well documented in association with such hosts. However, the smaller sampling of invertebrate-derived enterococci yielded highly diverse and more rarely encountered species, including 18 new species either cultured directly from invertebrates or from the feces of vertebrate consumers of invertebrates. Compared to more extensively sampled vertebrates, it was estimated that less than 0.01% of the diversity of land arthropod host species has so far been examined for *Enterococcus* association (4). The extreme range of arthropod gut anatomies, physiologies, and diets (5), together with their tractability as model hosts, make them ideal systems for determining the principles that drive speciation as well as host-specific associations for microbes. Further, their occurrence in nearly all ecosystems makes them ideal for monitoring the mobilization and spread of antibiotic resistances in the environment.

Prior work found that enterococcal speciation paralleled the acquisition of carbohydrate utilization and amino acid metabolism pathways, likely matching the changing diet of new hosts (2). Aside from that, little is known about the drivers of speciation or host association, which undoubtedly include exposure to naturally occurring antimicrobial substances in the host diet as well as diverse host defenses. Some enterococcal species, most notably *E. faecalis,* are found across a wide variety of hosts, while other species appear to have evolved highly specialized associations so far restricted to a single host species (4). The genetic and mechanistic bases for these differences remain to be determined.

This study aimed to directly test the prediction that substantial unidentified *Enterococcus* species diversity—potentially including an implied vast number of genes well tuned for expression in the genetic context of an *Enterococcus* and potentially available to strains commonly infecting humans—exists in nature in the guts of arthropods and other invertebrates. Because of its lush natural biology, comparatively sparse population, and geographic isolation from large industrial-scale antibiotic application, the temperate Azorean Island of Terceira was chosen as the site for collecting and cataloging the diversity of enterococci associated with invertebrate hosts. This design also allowed us to detect signals of geographic isolation—allopatry versus sympatry—as a driver of enterococcal speciation. The nearest continental landmass is 1,400 km distant (6). The Azores emerged in the Atlantic Ocean as volcanic islands about 6 million years ago (7), recent in the projected 400-million-year evolution of the enterococci (2). If geographic isolation drove the evolution of enterococci, clusters of species related to one or a few founders seeded at the time Azores land first appeared would be predicted to be disproportionately represented.

To determine (i) whether arthropods and other invertebrates represented a large, unexplored reservoir of enterococcal genetic diversity and (ii) whether evidence that diversification is affected by geographic isolation can be found, two sets of samples were examined for their enterococcal content. First, control fecal samples derived from animals well sampled in other geographic locations, including mammals, reptiles, and birds, were examined to determine whether the enterococci associated with these hosts are similar to those isolated from other lands or anomalous as the result of geographic isolation. Second, enterococci associated with diverse arthropods and invertebrates, little studied anywhere, were examined for the extent or limitation of diversity and for their relationship to known species found elsewhere. The results support the existence of vast unexplored genetic diversity in enterococci inhabiting invertebrates, including entirely new species, and failed to support the competing hypothesis that the diversity found in this study stems from geographic isolation. In sum, even at unusual levels of geographic isolation, adaptation to host diet and gut physiology, as well as successful transmission, are the main drivers of *Enterococcus*–host association. Additionally, this large and well-defined collection of enterococcal isolates from insects and other invertebrates allowed the classification of *E. casseliflavus* and *E. flavescens* to be quantitatively assessed, resolving a long-standing controversy.

## RESULTS

### Collection of enterococci from diverse invertebrate hosts on Terceira Island

**Fig 1:**
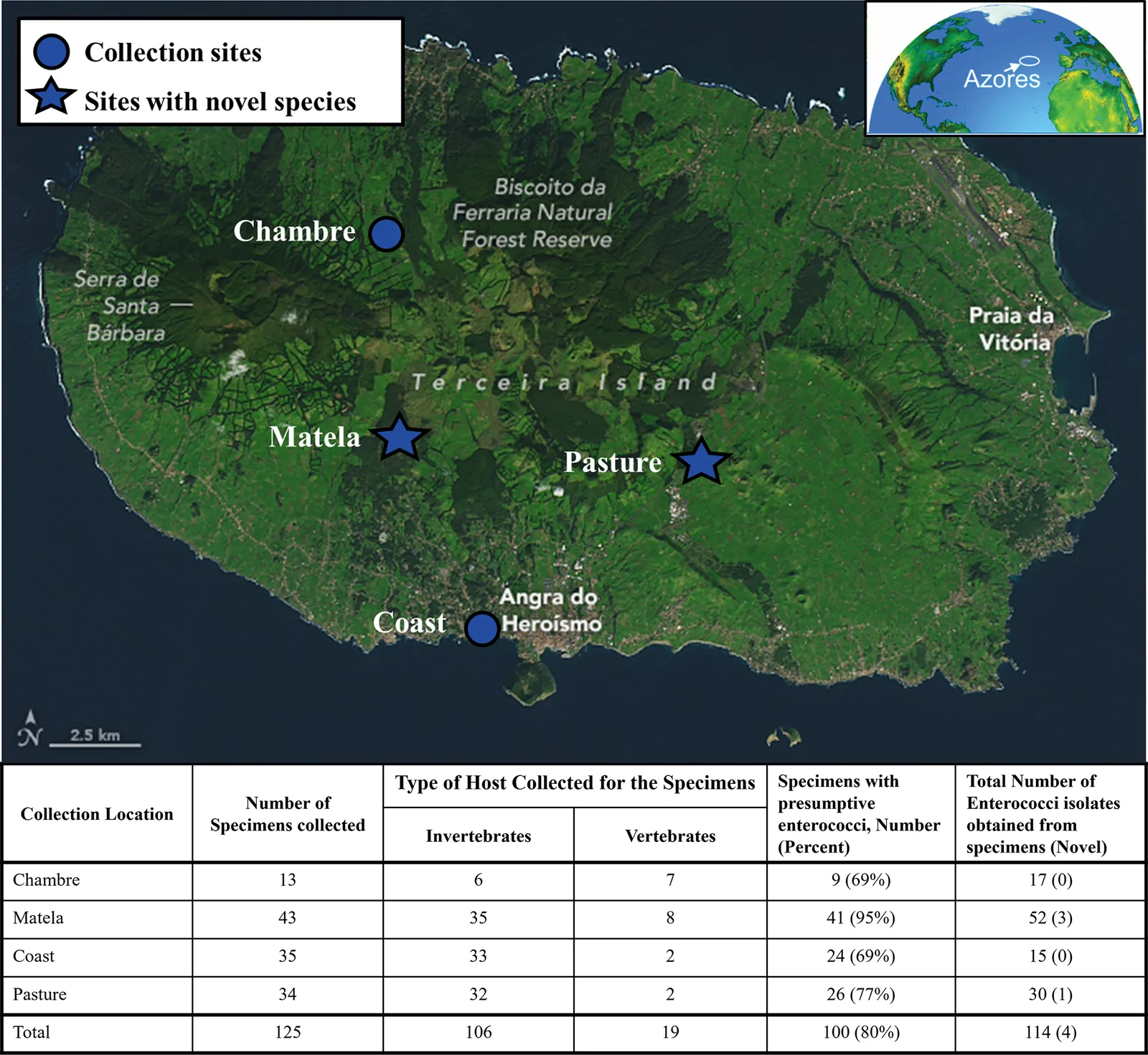
Sampling locations and specimens collected on the island of Terceira. Sampling locations are marked with blue circles or stars. Stars represent areas where specimens containing novel species of enterococci were collected. Chambre and Matela represent forest nature preserves. Pasture is an agricultural land use site managed by the University of the Azores. Coast indicates the coastal campus of the University of the Azores in an area of comparatively high human occupancy. (Imagery from NASA Earth Observatory.)

The Azorean island of Terceira was selected as the site for this study because of its recent emergence as a volcanic peak and its geographic isolation. Moreover, the conical shape of the island allowed various levels of the watershed to be sampled to detect the influence of human activity even on this comparatively natural and sparsely populated rural island: nature preserves near the pristine central plateau, a mid-level small-scale agricultural band, and the predominantly human-populated coastal region (Fig. 1).

To test whether enterococci from the Azores were similar to those derived from animals well sampled elsewhere, 19 control fecal samples from non-agriculture-associated wild vertebrates, including mammals (*n* = 10), birds (*n* = 7), and reptiles (*n* = 2) , were examined. Those specimens were plated on bile esculin azide selective medium, assessed by Gram stain, and examined phenotypically for catalase production and hemolysis on blood agar. The genomes of strains determined likely to be enterococci were then sequenced. High-quality, near-finished hybrid genome assemblies were generated using a combination of short- and long-read sequencing, and bona fide enterococci were obtained from 15 of 19 vertebrate samples (79%). Other sequences were identified as either stemming from mixed cultures that were not further resolved or as related Gram-positive bacteria, *Lactococcus* and *Staphylococcus*, capable of growing on enterococcal selective medium (8). For species identification, assemblies were compared against reference genomes from 99 known or proposed enterococcal species in NCBI using FastANI (9), with a threshold of 95% average nucleotide identity (ANI; the operationally defined species boundary).

To assess the existence of enterococcal diversity within invertebrates, and to detect the potential influence of geographic isolation on that class of hosts, a total of 106 invertebrate samples were collected from four sites representing different levels of human activity (Fig. 1). Invertebrate samples represented diverse taxa, including Insecta (*n* = 66), Diplopoda (*n* = 26), Crustacea (*n* = 5), Annelida (*n* = 4), Chilopoda (*n* = 3), and Arachnida (*n* = 2). Herbivorous insects were the most abundant and diverse hosts sampled (*n* = 59), followed by carnivores (*n* = 14), saprophages (*n* = 6), and omnivores (*n* = 3). From the invertebrate specimens, 42% (44 of 106) yielded enterococcal species confirmed by sequence, in good agreement with the rate of recovery of enterococci by culture from insects historically (3).

Of seven species associated with vertebrates, *E. mundtii* was most abundant, found in nine of 15 (60%) of *Enterococcus*-positive samples, followed by *E. faecalis* (seven of 15; 47%) and *E. casseliflavus* (six of 15; 40%). This largely mirrored the relative abundance of species from a recent global study that did not include the Azores (4), where *E. mundtii*, *E. faecalis*, and *E. casseliflavus/E. flavescens* (combined in that study because of close relatedness) (4) constituted three of the four most commonly encountered species (Fig. S1). In the comparator study (4), *E. faecium* was the second most commonly isolated species from vertebrates and the fourth most common species in invertebrates. It was found in 2/15 Azorean vertebrate samples. The collection of the previous study was skewed toward vertebrate sampling, including agricultural and captive animals reared by humans (4), whereas those sampled here were from wild foraging non-captive animals. Nevertheless, there is extensive similarity in the species of enterococci derived from vertebrate fecal specimens in both studies and little signal of an effect of geographic isolation on the species of *Enterococcus* associated with vertebrate hosts.

**Fig 2:**
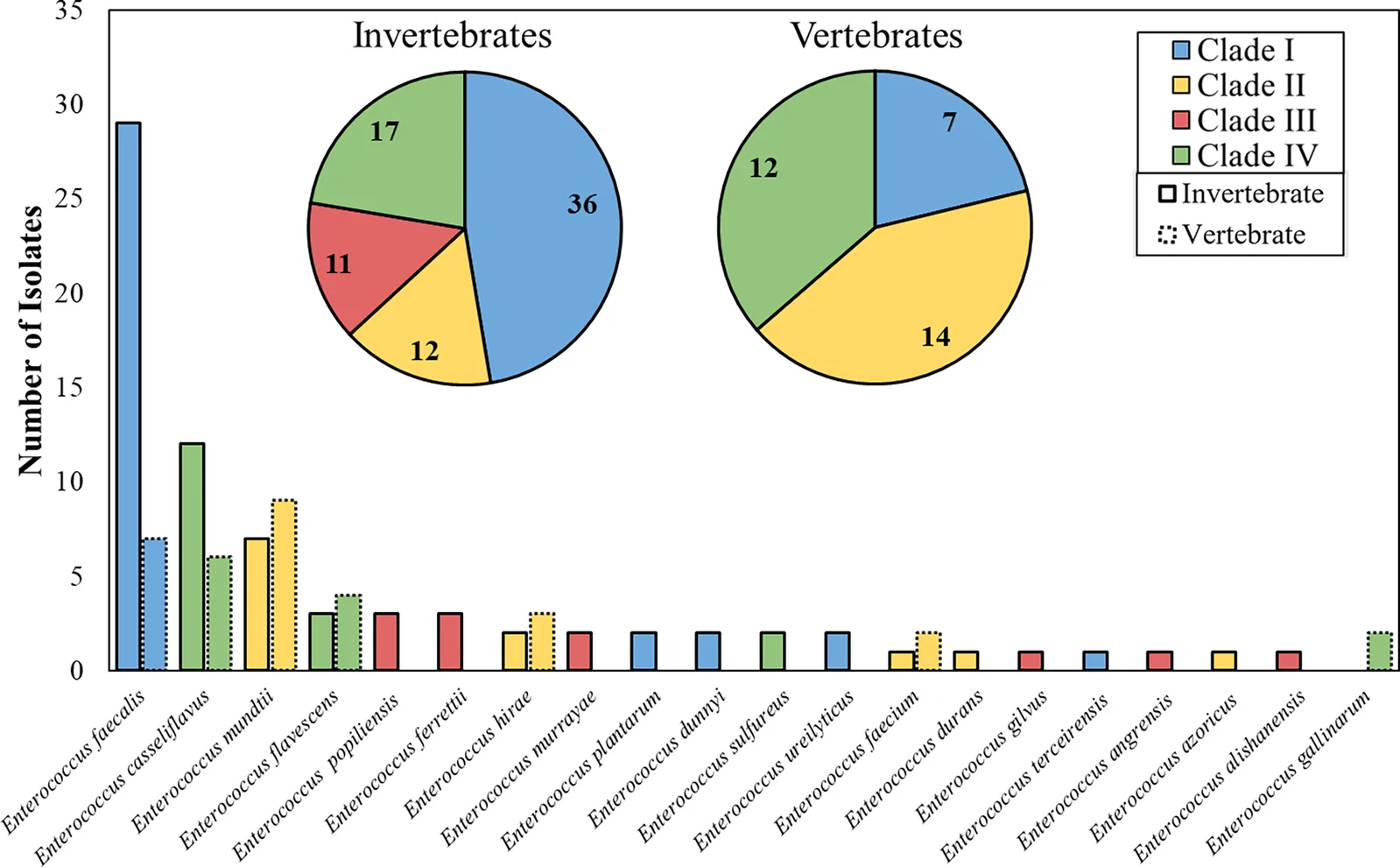
Clade representation and species abundances in vertebrate and invertebrate specimens. Bar graph representing the isolated species. Color represents clade of given species, and dotted bars represent vertebral origin of the isolates. Inset pie charts represent the number and percentage of isolates collected based on clade.

In contrast, enterococci from invertebrate hosts were identified as belonging to 19 species, with 14 exclusive to the invertebrate samples. This represents a Shannon diversity index for invertebrates of *H* = 2.23 compared to *H* = 1.81 for vertebrate controls. At the time of collection, these represented 15 known species and five novel species (with novel species being defined as possessing <95% nucleotide sequence similarity across shared genes [9] with any previously described species of *Enterococcus* [10]) (Fig. 2). One novel species was subsequently characterized at the genome sequence level and published as *Enterococcus alishanensis* (11), leaving four novel species identified here (Table 1). *E. faecalis*, found previously to be the most commonly encountered generalist across all hosts (4), was by far the most abundant species recovered from invertebrate samples (66%, 29 of 44), followed by *E. casseliflavus* (27%, 12 of 44) and *E. mundtii* (16%, 7 of 44). These three species made up about two-thirds of all isolates found for both vertebrates (67%, 22 of 33) and invertebrate (63%, 48 of 76) specimens, likely stemming from connectedness through the food chain (Fig. 2). Interestingly, among invertebrate samples, *E. faecium* was only isolated once from an omnivorous/caprophagic fly (4). *E. gallinarum* was the only species exclusively found in control vertebrate samples, with one isolate from bird and one from mammal feces. Phylogenetically, species of *Enterococcus* Clade III (6/19), followed by Clades I and II (5/19 each) and Clade IV (3/19) were found in association with invertebrates. In contrast, vertebrates more often yielded isolates of Clades II and IV (3/7 each) and Clade I (1/7), with none yielding a Clade III isolate. This suggests that either invertebrates colonized by Clade III strains are rarely consumed by the vertebrates studied or that they are transients that do not persist in the vertebrate digestive tract in numbers sufficient to be detected in these specimens.

**TABLE 1:** Candidate novel species described in this study^*a*^

Species name	Strain(s)	Clade	Most related species	ANI (%)	Genome size (Mb)	Host(s)	Sampling location	Unique OGs (no.)	Putative gene functions of unique gene functions (homolog)
*Enterococcus azoricus*	AZ194	II	*Enterococcus huntleyi*	80.8	3.64	European earwig	Pasture	25	Cytochrome C biogenesis (CcmF); vitamin B12 import (BtuD)
*Enterococcus popiliensis*	AZ128,AZ135, AZ186	III	*Enterococcus hulanensis* (*n* = 3)	83.1; 83.0; 83.0	4.75; 4.75; 4.7	Japanese beetle (*n* = 3)	Matela (*n* = 2); Pasture (*n* = 1)	28	Cobalamin-independent methionine synthase (MetH)
*Enterococcus angrensis*	AZ109	III	*Enterococcus pallens*	80.4	4.08	Armyworm moth	Matela	26	Polyketide biosynthesis (Snoal); L-Ala-D/L-glu epimerase (ycjG)
*Enterococcus terceirensis*	AZ126	I	*Enterococcus ureasiticus*	85.8	3.34	Earthworm	Matela	9	Endonuclease involved in phage immunity (ORF C62)

^*a*^ ANI quantifies distance to most closely related known species. Unique OGs (orthogroups) refer to genes that are not found elsewhere in the *Enterococcus* genus (further description in Data set S2). Homologs indicate proteins from other bacterial species related to those encoded by unique genes of novel *Enterococcus* species, identified based on conserved structures determined using Phyre2 (12).

We next quantified the relative contributions of vertebrate- and invertebrate-derived *Enterococcus* strains to the diversity of genes present in enterococci—total genetic richness. Using OrthoFinder, 14,069 unique orthogroups were found in all enterococcal genomes from this study. Of those, 1,027 were shared by all strains, and an additional 5,360 occurred in at least one strain from both a vertebrate and an invertebrate, together representing 45% of the unique orthogroups identified in enterococci in this study. Only 6% of the total orthogroups were exclusive to vertebrate-derived enterococci (*n* = 892), whereas 48% were exclusive to invertebrate-derived enterococci (*n* = 6790), representing the largest category of all enterococcal genes examined. This high level of genetic richness in invertebrates likely reflects both the extensive anatomic diversity of digestive tracts and the range of dietary substrates they consume.

### Phylogenetic placement of Azorean strains

**Fig 3:**
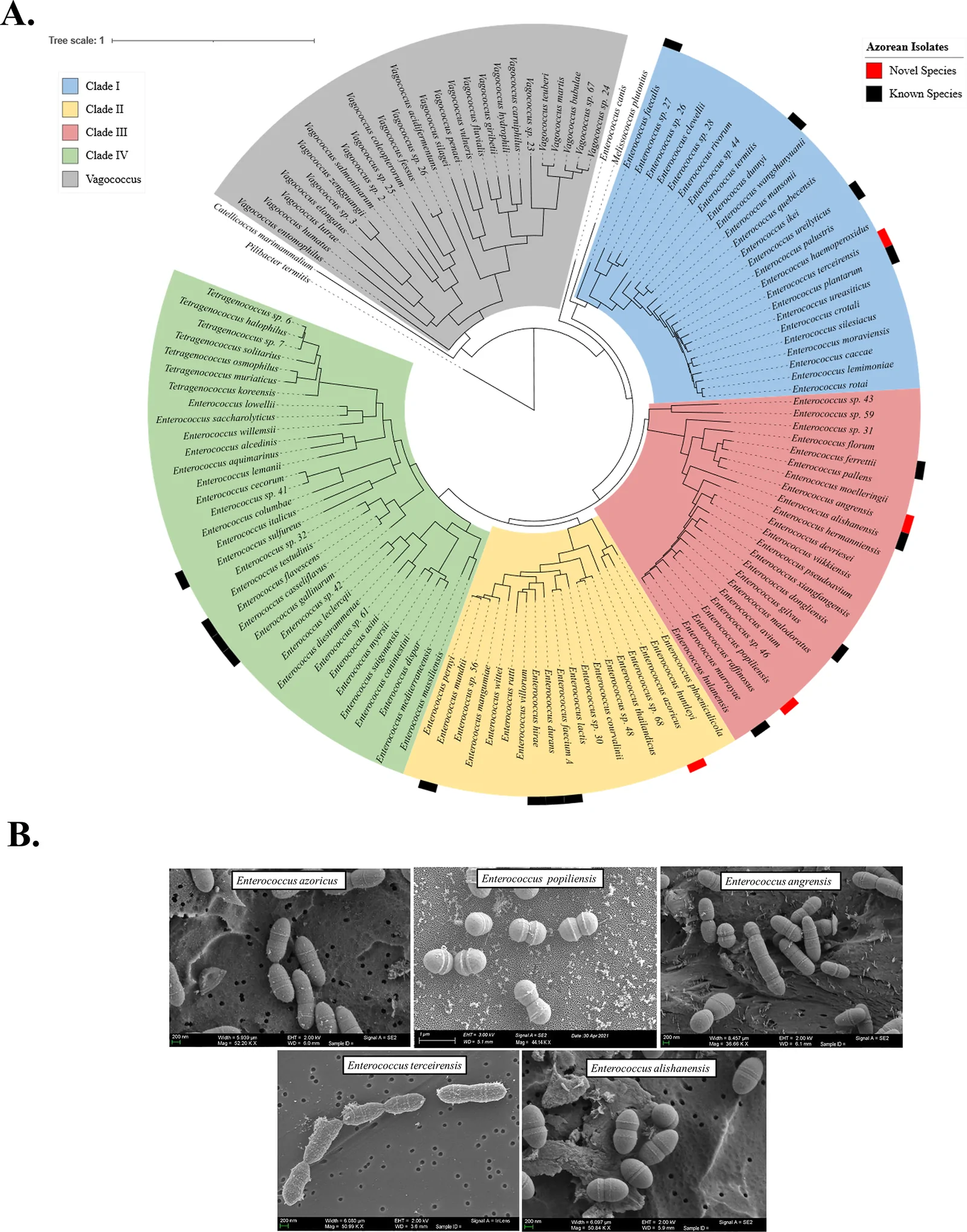
Phylogeny and morphology of novel species collected from the Azores. (**A**) Phylogenetic tree constructed from EnteroGenomeDB. Species isolated in this study are designated by black bars, and novel strains are marked with red bars. (**B**) SEM images of four novel strains plus *Enterococcus alishanensis*.

To detect potential clustering of species stemming from geographic isolation, we next placed each Azorean isolate into the genus phylogeny (4). To do this, we first created a comprehensive database of enterococci, which we called EnteroGenomeDB (Materials and Methods). In brief, we downloaded all *Enterococcus* genomes from NCBI GenBank and combined these with all isolates sequenced in our prior studies, published (2, 4) and unpublished, for a total of 33,376 enterococcal genomes. We also included 109 *Vagococcus*, and two additional related outgroup genomes. Genomes were clustered using a 95% ANI threshold to classify discrete species, which yielded 103 ANI-identified species of *Enterococcus* and 26 species of *Vagococcus*. We selected a single representative from each, performed orthogroup clustering, and used single-copy core gene alignments to construct a phylogenetic tree using IQ-TREE (13). As expected (4), genomes clustered into four distinct clades representing ancestral divisions within the *Enterococcus* genus (Fig. 3A). Branches separating major clades were well supported, with bootstrap values greater than 85%. While the overall structure of the phylogenetic tree, consisting of four clades, was similar to our previous studies using fewer genomes (2, 4), the inclusion of additional isolates from NCBI and from Azorean invertebrates led to the transposition of the branch points of Clade II and Clade III, suggesting a potentially more ancient origin of Clade III.

The more basal placement of Clade III along with Clade I would account for their greater association with invertebrate- compared to vertebrate-derived samples, as invertebrates terrestrialized first (2). When examining the placement of the Azorean strains within this phylogeny, we observed that Clade I, the most basal clade that includes *E. faecalis*, made up a large plurality of isolates from invertebrates, representing nearly half of all enterococci isolated (36 of 76, 47%). In contrast, enterococcal isolates from vertebrates were dominated by Clade II (14 of 33, 42%), which has been shown in previous studies to have genomic evidence of a mammalian/synapsid adaptation signature (1) (Fig. 2). Importantly, enterococci from invertebrates spanned all four clades of the genus, with little evidence of clustering suggestive of a recent common ancestor driven by geographic isolation. Finally, in addition to the novel species identified among the Azorean isolates, 25 additional species were cataloged within the sequence data used to build the phylogeny (Fig. 3A) represented as a number preceded by “*Enterococcus* sp.,” “*Vagococcus* sp.,” or “*Tetragenococcus* sp.”. A more complete analysis of those genomes will be presented separately.

### Isolation of additional strains of recently discovered species

The identification among the Azorean collection of enterococcal species that are so rare as to have been only recently discovered elsewhere further discounts geographic isolation as a driver of host association in this sample set. Three of these species, *Enterococcus ferrettii*, *Enterococcus murrayae*, and *Enterococcus alishanensis*, are in Clade III, the clade only isolated from arthropods in our study. *E. ferrettii* and *E. murrayae* were both isolated from multiple Azorean insects, including from hoverflies and bees (*E. ferrettii*), and ground beetles and earwigs (*E. murrayae). E. ferrettii* and *E. murrayae* both were initially described as being isolated from Kemp’s Ridley sea turtles stranded off the coast of Massachusetts due to cold stunning. These turtles range widely across the Atlantic basin, including the Azores (14). They reside in coastal waters, and notably, their preferred food is arthropods, in particular crabs (14). The third Clade III species, obtained from a yellow fly (order Diptera) caught in the Matela Forest Nature Preserve on Terceira Island and now called *E. alishanensis*, was initially designated as a fifth novel species from this study at the time of collection in 2019 and initial report in 2021 (10). Since then, the genome sequence of *E. alishanensis*, originally isolated from fresh coffee cherries collected at a farm in the Ali Mountain region of Taiwan in 2014, was published (11). We therefore defer to that publication for species designation. The Azorean isolate of *E. alishanensis* was obtained from a yellow fly (order Diptera) caught in the Matela Forest Nature Preserve on Terceira Island.

The fourth recently described species, for which we found two representatives in the Azores, is *Enterococcus dunnyi*, a Clade I *Enterococcus* previously isolated from a ground beetle in Alabama (USA) and a captive African bullet cockroach. In the Azores, it was isolated from a European earwig (*Forficula auricularia*) and from a Devil’s Coach-horse beetle (*Ocypus olens*) (4). These observations highlight the cosmopolitan distribution of even rare enterococci globally.

### Characterization of novel species

Excluding *Enterococcus alishanensis,* the remaining four novel enterococcal isolates are taxonomically unique with percent ANI relatedness to their nearest known neighbors ranging from 78.4% to 85.8%, well below the 95% threshold. Using a previously published molecular clock, the distance between each new species and its nearest known sister species indicates a last common ancestor on a timescale of >50 million years ago (2), substantially exceeding the 6 million years since the emergence of the island chain. SEM images revealed that all novel species, including *Enterococcus alishanensis*, occur as diplococci when cultured and share an ovoid shape and size typical of the genus (Fig. 3B).

#### Enterococcus terceirensis

**Fig 4:**
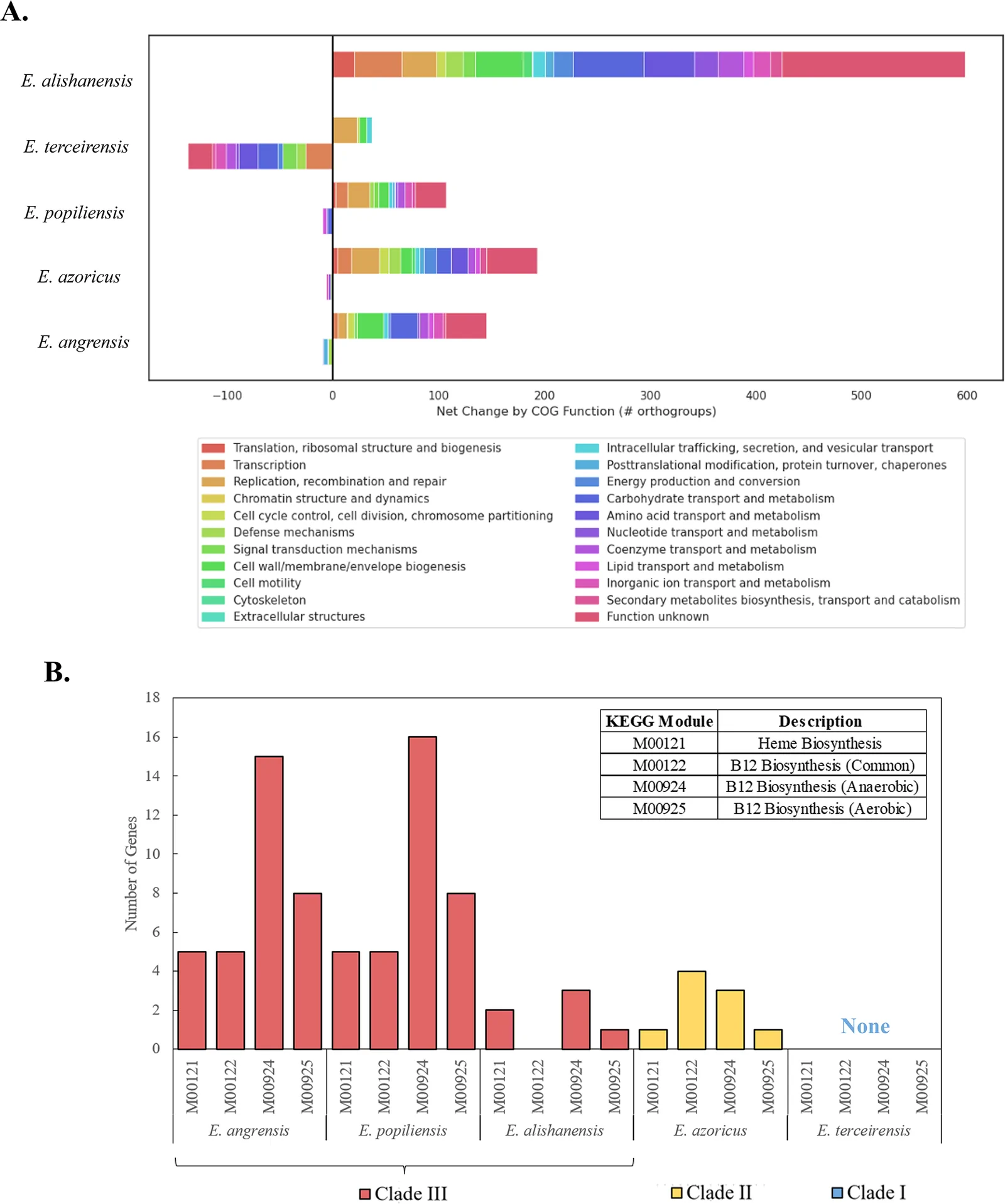
Characterization of genes from novel species collected from the Azores. (**A**) Net change in number of orthogroups gained and lost for a given COG grouping compared to the species’ respective nearest neighbor. (**B**) Vitamin B12 biosynthesis genes present in novel species and *E. alishanensis*. Colors represent the clade of organism, as in Fig. 3.

Novel candidate species *Enterococcus terceirensis* (Clade I) was identified from an annelid (earthworm) collected in the Matela Forest. Nearest neighbor analysis revealed it shares a most recent common ancestor with *Enterococcus ureasiticus* (85.7% ANI; Table 1), a species initially isolated from two well water samples in Canada (15) but of unknown host association. *E. terceirensis* possesses an approximately 10% smaller genome than *E. ureasiticus* (3.6 Mb *E. ureasiticus* versus 3.34 Mb *E. terceirensis*). *E. terceirensis* lacks 31 genes related to carbohydrate transport and metabolism that are present in its nearest taxonomic neighbor (Data set S1). This includes the absence of the ethanolamine (*eut*) and propanediol (*pdu*) utilization pathways, as well as the associated bacterial microcompartment proteins. High ethanolamine availability and utilization is strongly associated with animal hosts, particularly in the intestine, where it allows commensal organisms to derive energy from their host rather than rely on dietary carbohydrates (16). *E. terceirensis* also lacks the ability to synthesize vitamin B12, a key and energetically expensive cofactor in ethanolamine utilization (Fig. 4B).

#### Enterococcus azoricus

A novel Clade II species was also identified, named candidate species *Enterococcus azoricus*, which was isolated from a European earwig (*Forficula auricularia*) collected at the mid-island agricultural site. It shares 80.8% ANI with recently described nearest neighbor, *E. huntleyi*, which was isolated from insectivorous chicken feces (4). *E. azoricus*, along with *E. huntleyi* and *E. phoeniculicola* (isolated from the uropygial [preen] gland of wild red-billed wood hoopoes) (17), forms the earliest radiation from the base of Clade II, separating these three species from the larger cluster constituting the remainder of the clade. *E. azoricus* possesses a 3.64 Mb genome, making it one of the larger genomes of Clade II, but it is similar in size to the other species on this early branch—its nearest neighbor, *E. huntleyi* (3.5 Mb), and *E. phoeniculicola* (3.9 Mb, the largest Clade II *Enterococcus* described to date). *E. azoricus* possesses 762 genes not found in *E. huntleyi* and lacks 363 genes that are present in *E. huntleyi*. This net turnover of 401 genes reveals an altered strategy for carbohydrate acquisition and metabolism, potentially well matched to the gut of the European earwig, a generalist omnivore with an extremely flexible diet (18). In place of a large complement of genes with plant polysaccharide-degrading functions, such as glycosyl hydrolases targeting arabinose-containing polymers found in cellulose or receptors involved in sensing and importing plant-derived sugars, including *chvE* (19), *E. azoricus* is enriched in a diverse array of phosphotransferase systems, sugar interconversion enzymes, and components of central carbohydrate metabolism pathways predicted to facilitate the metabolism of diverse carbohydrates. The genome uniquely contains genes for the utilization of ethanolamine and propanediol, traits common among gut-associated bacteria (20), as well as an ANTAR-containing response regulator protein known to control ethanolamine utilization in *E. faecalis* (21). These are juxtaposed to the genes required for the biosynthesis of vitamin B12 necessary for these processes (Fig. 4B) (22). Together, these features suggest an enhanced tropism for the gastrointestinal tract of its host. *E. azoricus* also possesses a large gene cluster that appears to couple citrate metabolism with sodium transport and membrane energetics. CitM, a Na^+^-dependent dicarboxylate transporter, transports citrate complexed with divalent metals into the cell, where citrate lyase (*citCDEF*) converts citrate into acetate and oxaloacetate. Oxaloacetate is converted to pyruvate by oxaloacetate decarboxylase (*oadGAB*), a process that uses the energy from decarboxylating oxaloacetate to pump sodium ions out of the cell. This not only allows for growth on citrate as a sole carbon source—a pathway classically associated with anaerobic growth in gut environments—but also generates a membrane potential that may be useful in high-salt or alkaline environments, or under anaerobic or microaerophilic conditions (23, 24). Notably, *E. azoricus* also possesses a gene not found elsewhere in the *Enterococcus* genus that shares sequence identity with a known integral membrane protein CcmF (Data set S2), part of a complex that is important for the maturation of soluble cytochrome C, a protein important for adaptation to alkaline environments (25) such as the foregut of insects like the earwig (26). Taken together, *E. azoricus* seems to be specialized for microaerophilic, alkaline environments within its host gut, where it utilizes a diverse array of microbially or host-liberated carbohydrates, including free citrate and ethanolamine, potentially liberated through host epithelial cell turnover (16).

#### Enterococcus popiliensis

As noted, Clade III species were unique to isolates from invertebrates in this study and were also the source of a disproportionate number of novel species previously as well (4), highlighting the undersampling of this group of *Enterococcus* hosts. The first novel Clade III species identified in the present study, candidate species *Enterococcus popiliensis,* was isolated from three separate Japanese beetles (*Popillia japonica*) collected from multiple locations on the island (Matela Forest and University Pasture). This suggests possible host specificity or tropism for this beetle host. All share about 83% ANI with their nearest neighbor, *Enterococcus hulanensis. E. hulanensis* is one of four lactic acid bacterial strains isolated from Chinese traditional pickle juice made in China’s Heilongjiang province from naturally fermented cabbage (27), providing few clues to its host. *E. popiliensis* is almost equidistant to *Enterococcus raffinosus,* with which it shares 82.3% ANI; but based on phylogenetic branch lengths, it is farther away than *E. hulanensis*. Compared to this nearest taxonomic neighbor, *E. popiliensis* possesses a net addition of 307 genes, including a large number of unique genes (*n* = 106) associated with mobile elements such as transposases, integrases, or phage. Its genome contains mobile genetic element-dominated genomic islands likely encoding either remnants of a large plasmid inserted into the chromosome or an integrative conjugative element. *E. popiliensis* possesses three plasmids (70 kb, 14.5 kb, and 7.6 kb), two of which (70 kb and 14.5 kb) have few identifiable metabolic functions other than mediating horizontal transfer and are not found in its nearest taxonomic neighbor. Notably, *E. popiliensis* lacks a full CRISPR-Cas system as well as the toxin component of an abortive infection (Abi) system (AbiEii toxin) (28) present in *E. hulanensis*. Similar loss of CRISPR-Cas systems and subsequent influx of exogenous genetic material is believed to have been central to the evolution of MDR hospital-adapted lineages of *Enterococcus* (29, 30).

#### Enterococcus angrensis

The second novel species from Clade III, candidate species *Enterococcus angrensis*, was isolated from an adult armyworm moth (*Mythimna unipuncta*) found in the Matela Forest. It is most closely related to another recently described species, *Enterococcus moelleringii* (84.6% ANI), which was isolated from the Kemp’s Ridley sea turtle (4). *E. angrensis*, along with *E. moelleringii*, *E. ferrettii*, and *E. pallens* (a species of unknown natural host, but first isolated from peritoneal dialysate of a patient with peritonitis 31, 32) form a cluster that branches off the Clade III lineage early, leading to their diversification separate from the rest of the clade. Compared to its nearest taxonomic neighbor, *E. angrensis* possesses a large number of genes important for cell membrane and wall biogenesis. It also possesses a cluster of genes containing the full pathway for the biosynthesis of molybdenum cofactors, which is not present in closely related species. The loss of this pathway is seen in organisms more intimately tied to a specific host, suggesting that *E. angrensis* is more of a generalist (33). Compared to its nearest neighbor, it also possesses genes that are important for rhamnose and mannose metabolism, as well as the ability to ferment carbohydrates into butyric acid, a process commonly found in bacteria isolated from anaerobic environments (34). Similarly, *E. angrensis* possesses anaerobic carbon monoxide dehydrogenase, used in the Wood–Ljungdahl pathway to reduce CO_2_ to CO in the production of acetyl-CoA, another important adaptation to an anaerobic environment (35). Unique within the *Enterococcus* genus, *E. angrensis* possesses an epimerase with identity to *pgrR*, an *E. coli* enzyme involved in peptidoglycan recycling under nitrogen starvation conditions (36). *E. angrensis* possesses other genes suggesting a unique need for survival in nutrient-depleted conditions, including a homolog to *tauC*, a transporter in *E. coli* induced during sulfur starvation (37), and transport proteins for nitrate, sulfonate, and bicarbonate located within the same gene cluster. Taken together, *E. angrensis* seems to possess adaptations to anaerobic, nutrient-deficient environments.

### Genome analysis of *Enterococcus alishanensis*

As noted above, we independently isolated and characterized a specimen of the new species *Enterococcus alishanensis*, with core growth characteristics being as previously described (11), but little associated information on unique features of its genome. Compared to its nearest neighbor *E. hermanniensis*, *E. alishanensis* has a much larger genome size (3.8 Mb, compared to *E. hermanniensis* 2.6 Mb). *E. alishanensis* has a larger branch separation from its nearest neighbor than any of the other novel species. Among the genes that distinguish *E. alishanensis* from *E. hermanniensis* are 51 not found elsewhere in the *Enterococcus* genus (Data set S2). Notably, these include a homolog of glycosyltransferase-encoding *ykcB*, known to reduce susceptibility to vancomycin in *Bacillus subtilis* (38), and a gene predicted to encode a protein similar in structure to the translocase multidrug resistance-associated protein 4 (MRP4), which actively extrudes antibiotics including cephalosporins out of cells (39). Like *E. angrensis*, *E. alishanensis* possesses a large cluster of genes encoding the full pathway for the biosynthesis of molybdenum cofactors.

As noted in previous studies (4), novel species identified in Clade III possess the necessary genes for the anaerobic and aerobic biosynthesis of vitamin B12 (Fig. 4B). The presence of these genes in Clade III organisms has primarily been seen in species with large genomes, which is consistent with the larger genomes of both *E. angrensis* and *E. popiliensis* (4.1 Mb and 4.7 Mb, respectively). Consistent with this pattern, *E. alishanensis*, with its smaller genome (3.5 Mb), lacks both the anaerobic and aerobic B12 biosynthetic pathways.

### Quantification of the separation between *Enterococcus casseliflavus* and *Enterococcus flavescens*

Our sampling of enterococci from Azorean invertebrates included 27 isolates of *E. casseliflavus* and *E. flavescens*, which are of special interest because, along with the immediately neighboring species *E. gallinarum*, they have the unusual properties of possessing large vancomycin resistance and motility operons as core species traits (40). Additionally, because of their close relatedness, there has been substantial debate about the relationship and taxonomic classification of *E. casseliflavus* and *E. flavescens* (41). Identified as species in 1979 (*E. casseliflavus*) and 1992 (*E. flavescens*), genetic studies have shown minimal to no divergence in 16S rRNA (42) or manganese-dependent superoxide dismutase (*sodA*) (43), D-alanine–D-alanine ligase (*ddl*), and the vancomycin resistance gene *vanC* (44) gene sequences. Experiments using DNA-DNA hybridization (45) and pulse field gel electrophoresis also failed to show significant differences between the two organisms (46), raising questions about their relationship and validity of species designations. Through sampling of Azorean invertebrates and with advancements in technology, we sequenced the genomes of 27 independent isolates of *E. casseliflavus* (20 strains) and *E. flavescens* (seven strains), allowing quantitative assessment of distance by ANI (9) between all pairwise combinations of isolates. International reference strains of *E. casseliflavus* (ATCC 12755, NBRC 100478, and EC20) and *E. flavescens* (ATCC 49996) were included in the comparison.

**Fig 5:**
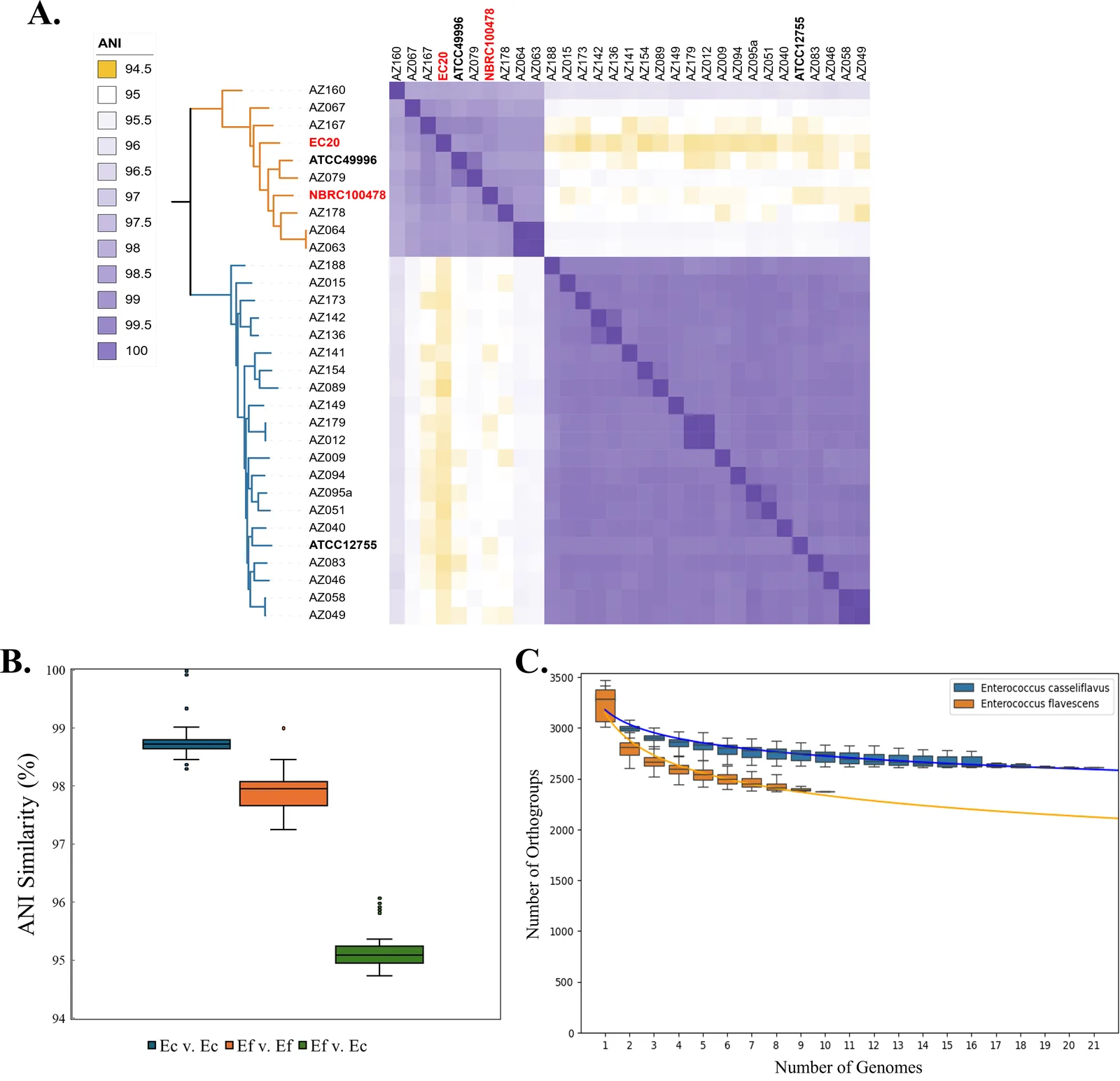
*E. flavescens* and *E. casseliflavus* have ANI differences at the border for species differentiation. (**A**) Heat map showing pairwise associations and hierarchical clustering of ANI between *E. casseliflavus* (20 strains) and *E. flavescens* (seven strains) isolates collected from the Azores, as well as reference strains for *E. casseliflavus* (ATCC 12755, NBRC 100478, and EC20) and *E. flavescens* (ATCC 49996). Strains listed in red are associated with inaccurate species designations in NCBI. (**B**) Box and whisker plot for pairwise differences in ANI between all intra- and interspecies combinations of *E. casseliflavus* (Ec) and all *E. flavescens* (Ef) strains analyzed in this study. (**C**) Curve showing the core genome of *E. casseliflavus* and *E. flavescens* as a function of the number of genomes analyzed.

The ANI difference between the ATCC type strains for the two species (ATCC 12755 and ATCC 49996) is 94.96%, barely below the 95% benchmark for species identity (47). Pairwise comparison of all strains from the Azores and reference strains revealed two distinct population clusters delineating *flavescens* from *casseliflavus* type organisms (Fig. 5A). ATCC type strains fell into their respective species groups. Interestingly, the widely used *E. casseliflavus* strains NBRC 100478 and EC20 both cluster within the *flavescens* group, indicating that these species along with their genome entries are currently misnamed and are potentially the source of some confusion. Within the two clusters, the average *intra*species ANI was 98.7% (range 98.3% to 100%) for pairwise comparisons within the *casseliflavus* group and 98% (range 97.3% to 100%) for the *flavescens* group. Pairwise comparison across the *flavescens* and *casseliflavus* groups shows an average *inter*species ANI of 95.2% (range 94.7% to 96.1%), straddling the 95% species threshold (47) (Fig. 5B), providing a whole-genome-based explanation for the controversy surrounding these species designations.

While comparing ANI of shared genes is useful to quantify the extent of sequence drift between two groups of strains, the gain and loss of genes that are not shared provide critical insights into the ecological drivers that divide populations and drive speciation events. Orthogroups exclusive to the core genomes (Fig. 5C) of either *E. casseliflavus* or *E. flavescens* were identified. *E. flavescens* strains in our set possess 1,450 unique orthogroups, compared to 1,056 orthogroups exclusive to *E. casseliflavus*. In contrast, 2,649 orthogroups are shared by both species (using an arbitrary threshold of present in >75% of strains to allow for both rare variants and incomplete genome sequences). To identify distinguishing features core to one species and not the other, we identified genes present in at least 75% of the genomes of one species that were rare or absent (present in 25% or fewer) in the genomes of the comparator species. This identified a set of 55 genes that broadly distinguish all or most *E. casseliflavus* from all or most *E. flavescens*, where they are largely absent, and 27 genes that conversely distinguish *E. flavescens* from *E. casseliflavus* (Table S1 and Fig. S3 through S5). Many of the distinguishing *E. casseliflavus* genes occur in large clusters and relate to survival functions such as cellular stress responses, metabolic flexibility, and protection against environmental factors. This includes a cluster of eight defense- and stress-related genes annotated as relating to the inhibition of lysozyme (*rsiV* and *pgdA*), providing broad substrate profile multidrug efflux (*mepA* and *mepR*), and an HchA-like chaperone that protects cells from severe heat shock, acid exposure, and starvation (48). A separately encoded group appears to provide additional protection from oxidative stress including genes related to the redox sensor and quinone reductase, *qorB*, followed by *mutT*, a versatile housekeeping enzyme which rids the cell of toxic nucleotide metabolites (49), and genes encoding both components of the ADP-ribosylation cycle (50), a multipurpose cellular process vital for the rapid response to oxidative DNA damage (51). A third cluster of genes provides metabolic versatility with a suite of genes involved in nutrient transport and salvage including an *araQ*-like arabinose transport gene, an *rihC*-like ribonucleoside catabolism gene, and a *dcuC*-like anaerobic transporter of fumarate, succinate, and malate (52). Taken together, the enrichment of genes in *E. casseliflavus* suggests that this cluster of strains routinely encounters both different stresses and different sources of nutrition than strains that cluster with *E. flavescens*.

*E. flavescens*, on the other hand, is enriched in all components of the glutathione uptake system, *ddpABCDF* (53), which is vital for protecting aerobic organisms from reactive oxygen species, suggesting that *E. flavescens* occupies a more aerobic niche (54). *E. flavescens* is also enriched in genes related to those for lactose (*lacEFG*) and maltose (*malH* and *glvC*) import and metabolism. Maltose is abundant in grain starch, and lactose is almost uniquely found in milk, adaptations that would seem to confer benefit in ecologies such as those of agricultural mammals. *E. flavescens* also uniquely harbors a gene encoding an EamA family efflux transporter most similar to YicL, which prevents the intracellular accumulation of toxic metabolites. It is located adjacent to that encoding RhaS, a transcriptional activator that in *E. coli* responds to the presence of L-rhamnose and upregulates genes for rhamnose catabolism (55).

**Fig 6:**
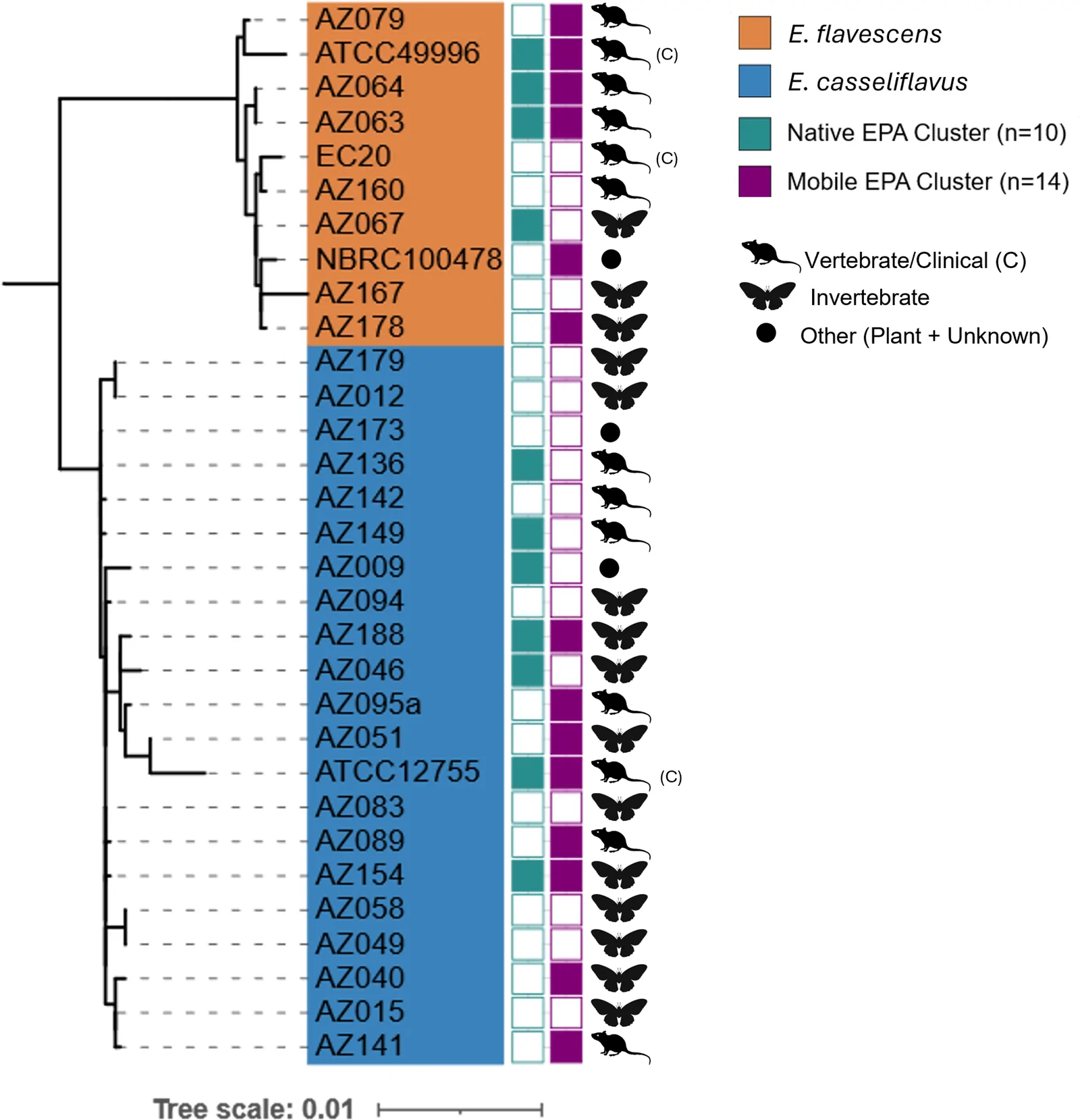
Comparison of EPA clusters in *E. flavescens* and *E. casseliflavus*. Phylogenetic tree constructed from *E. casseliflavus* (20 strains) and *E. flavescens* (seven strains) isolates collected from the Azores, as well as reference strains for *E. casseliflavus* (ATCC 12755, NBRC 100478, and EC20) and *E. flavescens* (ATCC 49996). Isolates are marked with colored squares representing EPA clusters present in their genome (lack of square represents no EPA gene cluster present in the strain’s genome), as well as the type of organism the strain was isolated from.

Most *Enterococcus* species possess genes for biosynthesis of a major rhamnose-containing cell wall polysaccharide (RhaCWPS) (56), originally termed enterococcal polysaccharide antigen (EPA) in *E. faecium (57*). Because of the association of YicL with RhaS, the functional importance of the RhaCWPS at the interface of Gram-positive bacteria and the host (58), and our previous finding of substantial variation in *epa* operons in Clade IV *Enterococcus* species including *E. flavescens* and *E. casseliflavus* (56), we specifically examined *E. casseliflavus* and *E. flavescens* genomes for differences in *epa* operons. Four genes (*rfbABCD*, homologous to *epaEFGH* in *E. faecalis*) are core for the synthesis of the rhamnose precursor and are broadly found throughout the *Enterococcus* genus. Interestingly, despite the fundamental importance of this cell wall polymer (56), these genes were observed to be variably present in strains of both *E. casseliflavus* (54%, present in 12 of the 21 genomes) and *E. flavescens* (70%, present in 7/10 strains) (Fig. 6). Further, the *rfbABCD* gene cluster was found in two different genomic contexts. The first appears to be the native gene cluster under the control of a LytTR family transcriptional regulator involved in cell wall turnover (59). This context occurred in 40% (4 of 10) *E. flavescens* and 29% (6 of 21) *E. casseliflavus* (Fig. 6). The second context in which *rfbABCD* is found is in association with a less homogenous group of genes that is universally enriched in transposases and integrases, suggestive of a mobile genetic element. This putatively mobile copy of *rfbABCD* is actually more common, occurring in 60% (6/10) *E. flavescens* and 38% (8/21) *E. casseliflavus* strains, with both types simultaneously occurring in 30% (3/10) of *E. flavescens* and 10% (2/21) of *E. casseliflavus* strains. Strains of both species that completely lack the rhamnose biosynthesis pathway have instead 11–15 genes that appear to encode an amino-sugar polymer adjacent to the universally conserved LytTR regulator. Further, strains possessing only the putatively mobile *rfbABCD* copy maintain the amino-sugar polymer-related operon at that site.

Because cell wall structures, including major carbohydrate components, occur at the host-microbe interface, we further examined the *rfbABCD* variability of strains with the host from which those strains derived. In general, it is more common for strains isolated from vertebrates to possess the *rfbABCD* operon than strains from invertebrates, with 77% of the former being positive compared to 40% of the latter (Fig. 6). This corresponds to 70% of *E. flavescens* isolates possessing *rfbABCD* operon with 60% of that species deriving from a vertebrate, compared to 57% of *E. casseliflavus* strains possessing the operon and 29% being isolated from vertebrate samples (Fig. 6).

## DISCUSSION

We are just beginning to understand the scope of diversity within the *Enterococcus* genus. In the 1970s, strains of both *E. faecalis* and *E. faecium* began to be isolated from human infections with antibiotic resistances conveyed by mobile elements (60). By the mid-1980s, resistance to last-line drugs compromised remaining bactericidal therapies (60, 61). The origins of these mobile elements and well-regulated resistance determinants (60) remain to be determined. We previously detected invertebrates as a potential source of enterococci possessing novel genes for adaptive traits to a wide range of host and environmental conditions (4). Here, we directly tested that prospect and found that even on a geographically isolated, generally rural and natural island, substantial species and genetic diversity exists in enterococci isolated from invertebrates, much higher than that present in association with comparatively heavily sampled vertebrates (4).

In 1972, Martin and Mundt isolated enterococci from 53% of insects sampled (3), but culture-based metabolic tests limited their ability to resolve species. Using genome sequence comparison technology, we identified 20 different enterococcal species—including four new species and four additional species only recently identified by us and others—from 106 enterococci isolated from invertebrate samples. Although yet to be systematically explored, this limited sampling implies a vast trove of genetic material optimized for regulated expression in a low G+C codon biased host background, which may well be the source of much of the DNA that has swollen the genomes of the CRISPR-deficient enterococcal lineages that have adapted to the hospital environment since the 1970s.

We previously showed that available nutrients, especially carbohydrates and amino acids, were major drivers of *Enterococcus* speciation, and that new species appeared to radiate in synchrony with the appearance of new hosts with new diets over the 400+ million years since the first terrestrialization of arthropods (2). The influence of other drivers of separation between bacterial populations that leads to the emergence of new species, including (i) biological drivers such as resistance to the innate gut defenses and physiologies in hosts ranging from invertebrates to mammals, (ii) geographical drivers such as the breakup of Pangea 200+ million years after enterococci emerged or the more recent emergence of isolated volcanic islands such as the Azores, and (iii) combined drivers of geology and biology that lead to highly adapted hosts with highly specialized diets, such as occurs on the Galapagos, has yet to be extensively explored. The opportunity to study enterococci in association with insects and other invertebrates on the Azorean island of Terceira provided a unique window to examine the diversity of enterococci associated with this class of hosts as well as the influence of geographic isolation.

Despite the remoteness of the sampling location, vertebrate-derived enterococci showed a species distribution similar to that found globally (4), indicating that neither geographic isolation nor the presence of unique sources of nutrition and competitors (62) are major drivers of host association for enterococci of those animals. Moreover, 80% (16/20) of the species we identified in association with invertebrates, including all of the most commonly isolated species, also occur elsewhere (4), including newly identified, rarely occurring species recently isolated from Massachusetts to Taiwan. Finally, even at the fine scale of intraspecies diversity, isolates of both *E. casseliflavus* and *E. flavescens* from Terceira Island nested on either side of the respective type strains with origins from other geographies (Fig. 6) (63–66). This provides further support that allopatric speciation stemming from geographic isolation is not a major driver of diversification and species radiation by enterococci.

Instead, what was found was extensive diversity among the enterococcal species isolated from invertebrates, which were largely isolated from soil-based ecosystems. The diversity of the isolated enterococci likely reflects adaptation to the extensive biological variation in invertebrate host diet with corresponding extensive variation in host gut physiology. In addition to the identification of four new *Enterococcus* species, the isolation of 27 strains of *E. casseliflavus* and *E. flavescens* allowed us to closely examine a speciation event in progress. Comparison of their genomes showed that they are precisely at the threshold identified by quantitative examination of many other bacterial species for being separate species (47), accounting for the controversy surrounding their separate species designations (41). Importantly, the average 5% nucleotide sequence divergence between shared genes of *E. casseliflavus* and *E. flavescens* is exactly the same as that we previously observed for the divergence between clades A and B strains of *E. faecium* (40). Extensive molecular clock analysis noted that divergence likely dated to an event a few thousand years ago, which was hypothesized to correspond to human urbanization and the expansion of agricultural practices. An analogous evolutionary split between human and bovine populations of *Staphylococcus aureus* was similarly dated to around 5,000 years ago (67). Gene signals identified here (e.g., unique gene gains for lactose and maltose fermentation) as being exclusive to *E. flavescens* are consistent with that hypothesis. Further, the recent movement of an insect-associated microbe into an agricultural animal gut may also have selected for cell wall adaptations that provide enhanced resistance to mammalian innate defenses, which could also explain the observed flux in the pathway encoding the major cell wall carbohydrate of these species. The precise role of the enterococcal Rha-CWPS in conferring resistance to host innate defenses, however, remains to be determined.

Almost half of the enterococci isolated from invertebrates were from Clade III, a clade that was notably absent in the vertebrate samples. Half of the novel species identified in this study were also from this clade. Clade III has many unique features setting it apart from the other clades, including the largest overall enterococcal genomes, up to 5.4 Mb, as well as the largest variation in genome size (2.6 Mb to 5.4 Mb, average of 4.4 Mb). A large subset of Clade III species, including the novel *E. angrensis* and *E. popiliensis* found our study, contains genes for the biosynthesis of cobalamin, or vitamin B12 (4), setting them apart from the enterococci of other clades where cobalamin auxotrophy is more common. Cobalamin is lacking in predominantly herbivorous diets, a common feature of invertebrates at the bottom of the food chain (68).

As noted previously (4), given the tremendous diversity in the gut physiologies, diets, and ecological ranges of the millions of terrestrial invertebrate host species that exist, and with only ~100 of those hosts ever having been sampled, there are likely to be vast numbers of new enterococcal species—and new genes well adapted to efficient regulated expression in the low G+C enterococcal background—yet to be found. The occurrence of the tightly regulated VanC-type vancomycin resistance operon as a core feature of the genomes of *E. casseliflavus*, *E. flavescens*, and *E. gallinarum*, a trait that remodels the peptidoglycan and otherwise carries a high fitness cost (69), is compelling evidence that, for at least thousands of years, isolates of these species have naturally encountered a glycopeptide-like selection at a frequency that favors maintenance of this operon in all representatives. Such selection could well occur in the gut of a detritivorous insect consuming decaying plant matter in the forest floor, precisely the substrate on which saprophytic, antibiotic-producing actinomycetes and streptomycetes thrive (70). If that is the case, then the vast genetic dark matter that this study shows to exist in enterococci inhabiting invertebrates in wide-ranging ecosystems, along with the high level of facility for genetic exchange that is characteristic of the enterococci (29), likely contributed to *E. faecalis* and *E. faecium* emerging among the vanguard of multidrug-resistant hospital pathogens in the 1970s and 1980s. Further studies will be needed to quantify this contribution and identify the mechanisms, selections, and circumstances that promote the movement of a potentially vast repertoire of enterococcal adaptive genes up and down the food chain.

The main limitation of this study is that it relied on initial cultivation for isolating prospective enterococci. The existence of novel species with unique nutrient requirements and atypical tolerance to components of selective media cannot be excluded and may have been overlooked. This highlights the importance of developing high-throughput culture-independent methods for rapidly assessing a specimen for enterococcal novelty. Nevertheless, even with this limitation ~4% of enterococci isolated from invertebrate specimens represented novel species, highlighting the extent of natural diversity in this host population yet to be discovered. Future work integrating culture-independent profiling, broader host replication, and functional testing of candidate niche-associated genes should further clarify how host ecology structures diversification across the *Enterococcus* genus.

### Conclusion

Enterococci—with genomes swollen by the accumulation of mobile elements (40)—likely emerged among the vanguard of multidrug-resistant hospital-adapted microbes that now constitute a leading global threat to healthcare, in part because of the ready availability of a large repertoire of still uncharacterized genes that permitted enterococci to adapt to and thrive in the guts of invertebrates in nearly all terrestrial environments over millennia. Continued exploration to define the drivers of host association, along with knowledge of that genetic repertoire, will illuminate the mechanisms and pathways that moved antibiotic resistance and other genes adapted in soil ecosystems into the modern hospital environment. That knowledge will guide strategies for limiting the transmission of new resistances and potentially also virulence traits (71, 72) and for quantifying the risk posed by currently unknown genes circulating in the enterococcal gene pool.

## MATERIALS AND METHODS

### Site description

Sample collection took place on the Azorean island of Terceira, a volcanic island with a subtropical climate located in the North Atlantic (38°43′40″N, 27°12′48″W). Terceira has an area of 400 km² and a human population of just over 50,000, who primarily live in a ring of urbanization encircling the volcanic cones at the center of the island. Sample collection was done at four ecologically diverse sites chosen to maximize the diversity of the insects and invertebrates collected (Fig. 1). These included two centrally located forested sites, both in protected areas of the Terceira Nature Park with minimal human interaction (Chambre and Matela), an agricultural pasture managed by the University of the Azores (Pasture), and a coastal shrubland habitat on the island’s southern coast, located near the capital city of Angra do Heroísmo (Coast).

### Sample collection and host identification

Insects and other invertebrates were collected either by direct sampling or by utilizing pitfall traps (Sampling permit CCPI: 39/2019/DRCT/ 59/2019/DRA). Traps were buried in the topsoil at least 1 m apart at the sampling sites and left overnight. Specimens were subsequently collected every morning from the traps. All invertebrate samples were photographed and identified to the highest taxonomic resolution possible, typically at the level of species. In addition to invertebrates, fecal samples from larger vertebrates were collected at sampling sites whenever encountered. All samples were transferred to sterile containers and either directly processed or kept at ˗20°C for 2–7 days until processed. All samples were collected from July to September 2019.

### Sample processing and isolation of enterococci

Enterococcal isolates were isolated in similar fashion as described previously (4). Briefly, invertebrates were mechanically disrupted and homogenized in sterile pH 7.4 phosphate-buffered saline. This slurry was plated onto bile esculin azide (BEA) agar (BD) and incubated aerobically for 72 h at 30°C. This temperature was chosen for incubation given that organisms were isolated from primarily ectothermic invertebrates and that this temperature was more biologically relevant to organisms from the island of Terceira in the Azores. Additional screening of colonies involved Gram staining and testing for phenotypes not characteristic of enterococci, including catalase production and hemolysis on blood agar. Colonies fulfilling the criteria for being presumptive enterococci were then re-streaked for isolation on fresh BEA plates and subsequently stored on brain heart infusion (BHI) agar (Biokar Diagnostics) slants for further analysis.

### Scanning electron microscopy

Scanning electron micrographs of novel species were generated by the Harvard Center for Nanoscale Systems. Bacterial cells were grown overnight in BHI and placed onto a 12 mm Nuclepore track-etch membrane filter (Whatman, Part no. 110405) or 13 mm Anodisc membrane filter (Whatman, Part no. 6809 7003), and Karnovsky’s fixative was used as the primary fixative. Following this, 1% OsO4 in Karnovsky’s fixative was used for post-fixation. Cells were then dehydrated, and the filters were dried in a Tousimis 931.GL critical point dryer. Using standard operation mode, the samples were sputter-coated in an EMS high-resolution sputter coater 150TS using 5 nm gold-palladium. The cells were imaged using a ZEISS Ultra-55 scanning electron microscope at 2 kV accelerating voltage. Approximate cell dimensions were obtained from SEM photos by comparison with the scale bar.

### Genome sequencing and assembly

Genomic DNA was extracted using a QIAamp 96 DNA Blood Kit (Qiagen) following the manufacturer’s instructions, with the addition of an initial digestion step with mutanolysin (Sigma Aldrich) to disrupt the recalcitrant cell wall of *Enterococcus*. The isolated genomic DNA was used to generate both short- and long-read sequencing in order to generate high-quality hybrid assemblies. For short-read sequencing, Nextera XT libraries were generated and sequenced with 150 bp paired-end reads on an Illumina NovaSeq. For long-read sequencing on an Oxford Nanopore GridION machine, libraries were constructed with the Oxford Nanopore SQK-LSK109 kit. All genome sequencing was carried out at the Broad Institute of MIT and Harvard University. Hybrid assemblies were generated using a combination of both long- and short-read data, as before (73).

### Construction of EnteroGenomeDB: the largest database of sequenced enterococcal genomes to date

In order to place the newly sequenced genomes in the context of previously identified enterococcal species, we constructed the largest enterococcal genome database to date, which we named EnteroGenomeDB. As of the writing of this manuscript, this database includes representatives of 99 enterococcal and 26 vagococcal species available in NCBI. We constructed this in two phases, including an initial download of 18,015 Enterococcaceae (taxID 81852) genomes from NCBI in May 2021, and an update of 18,969 additional genomes in October 2024, for a total of 36,894 genomes. We retained genomes that passed quality control filters using CheckM version 1.2.3 (74), including checks for contamination (<2% in the first phase and <5% in the second phase) and completeness (>90%). We also removed 550 that corresponded to metagenome-assembled genomes (MAGs). After all filtering steps, we were left with a total of 33,485 genomes.

After the initial download, we calculated all-vs-all ANI distances using FastANI version 1.33 (9) and performed hierarchical clustering using a distance threshold corresponding to 95% ANI (47), the working definition of a species. For each cluster, we selected a representative genome, selecting the highest-quality genome (low number of contigs, high N50, most complete). After the second download in 2024, we compared each newly downloaded genome against our existing species reference genomes, assigning each to an existing species if they matched with ANI > 95%. Only 42 genomes from the second download did not match existing species from the initial download; for these, we performed all-vs-all comparisons using MASH version 2.3, then performed hierarchical clustering using SciPy (version 1.15.3, scipy.cluster.hierarchy function) to form clusters with a maximum MASH distance of 0.05 between genomes. We identified 29 additional clusters among these newly downloaded genomes. The final data set of 34,485 genomes was grouped into 99 *Enterococcus* and 26 *Vagococcus* species.

### Comparative genome analysis

All genomes, including newly sequenced genomes and NCBI genomes, were uniformly annotated using Prokka (version 1.14.6), including a data set of manually curated, *Enterococcus*-specific genes (75). We performed additional functional annotation using eggNOG-mapper (version 2.1.12, database version 5.0.2, using the DIAMOND approach), including KEGG and COG annotations (76).

For each newly sequenced genome, we determined a species designation by comparison to the reference genomes in EnteroGenomeDB, using FastANI. Strains with ANI >95% were interpreted as belonging to the same species. The four newly sequenced strains with ANI <95% to all known species were designated as novel species.

To calculate a phylogeny of all enterococcal and vagococcal species, we first performed orthogroup clustering using OrthoFinder version 2.5.2 (77), using a single representative for each species, including newly sequenced genomes and EnteroGenomeDB genomes (total of 103 *Enterococcus* and 26 *Vagococcus*). We also included outgroup reference genomes for *Pilibacter*, *Catellicoccus*, and *Vagococcus*. We identified orthogroups that contained single copies of genes from at least 95% of all species to construct a phylogeny. We performed alignments of individual orthogroups using MAFFT (version 7.525 with default settings) (78). After concatenating the individual alignments, we inferred a phylogeny using IQ-TREE (version 2.2.5, using ModelFinder mode with 1,000 ultrafast bootstraps) (79). We rooted the tree using *Pilibacter* as the outgroup in iTOL (version 7) (80).

To validate the topology of the phylogeny inferred from core orthogroups and exclude any effects from horizontal gene transfer, we also calculated a phylogeny using AMPHORA2 (version 2.0) marker genes (81). We aligned marker gene sequences using MUSCLE (version 5.3) and filtered with Gblocks (version 0.91b), retaining the 30 marker genes present in >99% of genomes (82, 83). We built a phylogenetic tree using IQ-TREE (version 2.4.0)(79).

### Analysis of novel species

In order to identify features that distinguished each of the four novel species, we compared each to its nearest phylogenetic neighbor. Nearest neighbors were identified by calculating the shortest branch distance (DendroPy version 5.0.6, using the phylogenetic_distance_matrix function). We identified orthogroups present in each species, but not its nearest neighbor, and vice versa. Orthogroups gained or lost relative to their nearest neighbor were clustered based on genomic proximity, with a minimum cluster size of 10 genes and a maximum gene gap of two genes. Once clusters were selected based on these criteria, genes with gaps of up to five genes were added back to the clusters to improve coverage at the ends. Prophage sequences detected with PHASTEST were removed from these clusters.

Clusters in Azores genomes were assessed to determine whether they were chromosomal or plasmidic. All regions in the hybrid Illumina-Oxford Nanopore assemblies that were circular and smaller than 250 kbp were labeled as plasmids. Plasmer (version 23.04.20, database version 11/142023) and PlasmidFinder2 (software version 2.0.1, database version 1/18/2023) identified no additional plasmids (84, 85).

Within these clusters, we identified biosynthetic pathways differing between each pair, based on Prokka and eggNOG annotations. Annotation with BlastKOALA (kegg2 and PFAM database version 1/16/2018, HMMER version 3.2.1) was also performed. We visualized the presence of genes in pathways using the KEGG Color Mapper tool (https://www.genome.jp/kegg/mapper/color.html).

For each novel species, we also identified orthogroups containing genes not found in any other *Enterococcus* species. We confirmed that these were truly unique to the species by using BLASTP (version 2.2.30, -task “blastp” flag) to compare against all genomes in the EnteroGenomeDB database. Unique genes did not have any matches to any other *Enterococcus* genomes (E-value < 10e^−5^, % identity > 30%, and query coverage > 70%). For the unique genes with no known annotation, we used Phyre2 (version 2.2) to obtain annotations based on structural alignments (12, 86). To assess the potential for these novel genes to have been obtained by horizontal gene transfer, we examined their proximity to mobile genetic elements, including phage, plasmid, and insertion sequences (IS elements). PHASTEST (version 3.0) was used to identify prophage sequences (87), and BLASTN (version 2.12.0, -evalue 10 -word_size 11 -gapopen 5 -gapextend 2 -reward 1 -penalty 3) was used against a combined ISFinder and TnCentral database created on 10/7/2024 to identify insertion sequences and transposons. Insertion sequences were noted if they were present within the novel gene or within the 5 kb flanking regions. In order to examine the presence of vitamin B12 pathways across the novel organisms, we examine genes that were assigned to the following B12 synthesis-related KEGG modules: M00121 (heme biosynthesis, plants and bacteria, glutamate => heme), M00122 (cobalamin biosynthesis, cobyrinate a,c-diamide => cobalamin), M00924 (cobalamin biosynthesis, anaerobic, uroporphyrinogen III => sirohydrochlorin => cobyrinate a,c-diamide), and M00925 (cobalamin biosynthesis, aerobic, uroporphyrinogen III => precorrin 2 => cobyrinate a,c-diamide).

### Comparison of *E. casseliflavus* and *E. flavescens* genomes

In order to investigate differences in gene content between members of these two species, we identified orthogroups using OrthoFinder version 2.5.4 across a set including four reference genomes downloaded from NCBI and 31 Azores genomes (10 *E. flavescens* and 21 *E. casseliflavus* genomes assembled from Illumina reads), as well as one *E. gallinarum* outgroup (77). We identified enriched orthogroups, defined as being contained in ≥75% of one species and ≤25% of the other.

We clustered the enriched orthogroups (57 in *E. casseliflavus* and 28 in *E. flavescens*) based on their genomic positions. As we used the more contiguous hybrid assemblies to determine genomic synteny, we excluded two very short orthogroups from the Illumina assemblies that were not annotated as genes in the hybrid assemblies, leaving a total of 83 enriched orthogroups for downstream analysis. As most enriched orthogroups clustered similarly across all strains, with a location within two genes of another enriched orthogroup, we grouped orthogroups that were within 10 genes of each other across 75% of the strains in which they were present, in order to capture all clustering. To compare the synteny of clustering across strains, we selected representative genomes for each species (*E. casseliflavus* AZ179 and *E. flavescens* AZ064) to compare against. For the single orthogroup not found in *E. casseliflavus* AZ179, we used *E. casseliflavus* AZ136 as a reference.

To annotate each cluster, we obtained COG and KEGG annotations for each gene, including KEGG metabolic pathway membership, by submitting proteomes to eggNOG-mapper (emapper version 2.1.12) to get annotations including COG, KEGG, and Pfam. For each cluster where a majority of members shared COG functions, we assigned cluster-level functional annotations including (i) metabolism, (ii) transport, (iii) cell wall biogenesis, and (iv) cell motility. To identify antimicrobial resistance (AMR) genes, we ran AMRFinderPlus (version 3.11.2, database version 2022-12-19.1,-0 -Escherichia --plus). For genes annotated as hypothetical by Prokka, we ran BLASTP against the ClusteredNR database and used PHYRE2.2 (confidence >70%) to search for structural homology. GhostKOALA (version 3.1) was also used to provide additional annotations to validate key biosynthetic pathways differing between the two species. KEGG-Color Mapper was used to visualize these results, and some manual curation was performed based on genomic context.

To identify mobile elements near clusters, we searched for prophage sequences using PHASTEST (version 3.0) on the selected representative genomes and assessed their overlap with our clusters. Insertion sequences and transposons were identified using BLASTN (version 2.12.0, -evalue 10 -word_size 11 -gapopen 5 -gapextend 2 -reward 1 -penalty 3) by searching against a database created on 10/07/2024 from downloads of the ISFinder and TNCentral databases (88). Insertion sequences were mapped against enriched genes, and any overlap within a gene or within the 5 kb flanking regions was labeled as a hit.

We also examined *EPA* genes in more detail in these *E. casseliflavus* and *E. flavescens* genomes, focusing on seven key genes from *E. faecalis* V583 (GCA_000407305.1). We searched for *epaB-H* using BLASTP (version 2.12.0), with thresholds of percent identity ≥70% and query coverage ≥80%.

A phylogenetic tree of the *E. casseliflavus* and *E. flavescens* genomes, based on Amphora marker genes, was built using FastTree (55) and rooted with the *E. gallinarum* outgroup. To perform a detailed investigation of the divergence between *E. flavescens* and *E. casseliflavus*, we calculated all-vs-all ANI values between all newly sequenced Azores assemblies from these two species, as well as four previously sequenced reference genomes, using FastANI (9), and visualized this alongside the phylogeny of these strains.

## SUPPLEMENTAL MATERIAL

Fig. S1 mbio.01234-26-s0001.tif *Enterococcus* isolates per species from vertebrates.

Fig. S2 mbio.01234-26-s0002.tif *Enterococcus* isolates per species from invertebrates.

Fig. S3 mbio.01234-26-s0003.tif Genome sizes for *casseliflavus* and *flavescens*.

Fig. S4 mbio.01234-26-s0004.tif *casseliflavus* and *flavescens* pan-genomes.

Fig. S5 mbio.01234-26-s0005.tif *casseliflavus* and *flavescens* orthogroup enrichment.

Fig. S6 mbio.01234-26-s0006.tif Novel species orthogroup enrichment.

Supplemental material mbio.01234-26-s0007.docx Supplemental tables and figure legends.

Data set S1 mbio.01234-26-s0008.xlsx Nearest neighbor analysis for novel species.

Data set S2 mbio.01234-26-s0009.xlsx Nearest neighbor analysis for novel species.

ASM does not own the copyrights to Supplemental Material that may be linked to, or accessed through, an article. The authors have granted ASM a non-exclusive, world-wide license to publish the Supplemental Material files. Please contact the corresponding author directly for reuse.
