# Supplementary material for: Novelty, diversity, and genetic dark matter in enterococci of invertebrates
Source: mBio. 2026 Aug 20;17(9):e01234-26. doi: 10.1128/mbio.01234-26 (PMC13556245; doi:10.1128/mbio.01234-26)
Supplement: Supplementary file 1 [file mbio.01234-26-s0007.docx]

Supplemental Information for:

***Novelty, diversity and genetic dark matter in enterococci of invertebrates, including quantitative resolution of E. casseliflavus from E. flavescens***

Bruna F. Sgardioli^1,2^*, Matthew C. Phillips^3,4^*, Arjun Miklos^5^, Kailey Fleiszig-Evans,^5,7^ Abigail L. Manson^5^, Suelen Scarpa de Mello^4,7^, Terrance Shea^5^, Aysun Urhan^5,8^, Ryan Whipple^4,7^, Rauf Salamzade,^5,6^ Jasper Sanders^5^, Paulo A. V. Borges^9^, Maria de Lurdes Nunes Enes Dapkevicius^1,2‡^, Ashlee M. Earl^5‡^, Michael S. Gilmore^4,7‡^

1. Faculty of Agricultural and Environmental Sciences, University of the Azores, 9700-042 Angra do Heroísmo, Portugal
2. Institute of Agricultural and Environmental Research and Technology (IITAA), University of the Azores, 9700-042 Angra do Heroísmo, Portugal
3. Division of Infectious Diseases, Department of Medicine, Massachusetts General Hospital, Boston, MA 02114
4. Harvard Medical School, Boston, MA 02115
5. Infectious Disease and Microbiome Program, Broad Institute, Cambridge, MA 02142
6. Department of Medical Microbiology and Immunology, School of Medicine and Public Health, University of Wisconsin- Madison, Madison, WI 53706
7. Department of Ophthalmology, Mass Eye and Ear, Harvard Medical School, Boston, MA 02144
8. Delft Bioinformatics Lab, Department of Intelligent Systems, Delft University of Technology, Delft 2628XE, The Netherlands
9. University of Azores, CE3C—Centre for Ecology, Evolution and Environmental Changes, Azorean Biodiversity Group, CHANGE —Global Change and Sustainability Institute, School of Agricultural and Environmental Sciences, Rua Capitão João d’Ávila, Pico da Urze, 9700-042 Angra do Heroísmo, Azores, Portugal

* BFS and MCP contributed equally to this work

‡Corresponding authors: Michael S. Gilmore, Email: [michael_gilmore@meei.harvard.edu](mailto:michael_gilmore@meei.harvard.edu); Ashlee M. Earl, Email: [aearl@broadinstitute.org](mailto:aearl@broadinstitute.org); Maria de Lurdes Nunes Enes Dapkevicius, Email: [maria.ln.dapkevicius@uac.pt](mailto:maria.ln.dapkevicius@uac.pt)

**Supplemental Figures:**

**Supplemental Figure S1.** Comparison of Enterococcus isolates isolated from vertebrate hosts from this study versus Schwartzman et al., 2024. Number of Enterococcus isolates per species from vertebrates are shown from a) Schwartzman et al., 2024 (*4*), and b) from Azorean specimens in this study.

**Supplemental Figure S2.** Comparison of Enterococcus isolates isolated from invertebrate hosts from this study versus Schwartzman et al., 2024. Number of Enterococcus isolates per species from invertebrates are shown from a) Schwartzman et al., 2024 (*4*) and b) Azorean invertebrates

**Supplemental Figure** **S3**: Genome size for isolates of *E. flavescens* and *E. casseliflavus* from this study

**Supplemental Figure S4**: Pan-genome curves for *E. flavescens* and *E. casseliflavus*

**Supplemental Figure** **S5**: Functions of orthogroups enriched in *E. casseliflavus* and *E. flavescens*. A) Percentages of enriched orthogroups by COG; B) numbers of enriched orthogroups for each COG function

**Supplemental Figure S6**: Presence of COG annotations for orthogroups gained and lost in relation to the nearest neighbor for each novel species and *Enterococcus alishanensis*

**Supplemental Tables:**

Table S1: Orthogroup s enriched in *E. flavescens* (A) and *E. casseliflavus* (B). Groups of shaded rows represent syntenic clusters of genes (see methods).

A. Orthogroups found in ≥75% *E. flavescens* genomes (n=10), and ≤25% of *E. casseliflavus* genomes (n=21)

| Gene identifier | Putative Gene Name | Annotation | COG | Functional consensus for gene cluster |
| --- | --- | --- | --- | --- |
| AZ064_00220 | *lysM* | Peptidoglycan binding protein | M |  |
| AZ064_00476 | *-* | Hypothetical protein |  |  |
| AZ064_00649 | *-* | Nitrogenase, subunit molybdenum-iron protein alpha |  |  |
| AZ064_00669 | *adhB* | Zinc containing alcohol dehydrogenase | E |  |
| AZ064_01062 | *-* | MFS transporter | G | Carbohydrate transport & metabolism |
| AZ064_01063 | *malH* | Maltose-6'-phosphate glucosidase | G |  |
| AZ064_01064 | *glvC* | Maltose and glucose-specific phototransferase system, components EIICB | G |  |
| AZ064_01473 |  | Hypothetical protein |  |  |
| AZ064_01492 |  | Integral membrane protein | S |  |
| AZ064_01511 |  | Hypothetical protein |  |  |
| AZ064_02382 |  | 5'-deoxynucleotidase | K |  |
| AZ064_02444 | *rhaS* | HTH-type transcriptional activator | K | - |
| AZ064_02445 | *yicL* | Putative inner membrane transporter | EG |  |
| AZ064_02605 | *-* | Heme exporter | S |  |
| AZ064_02720 | *-* | Type II toxin-antitoxin system HicB family, antitoxin component | S |  |
| AZ064_02805 | *lacE* | Lactose-binding protein | G | Inorganic ion transport & metabolism |
| AZ064_02806 | *lacF* | Lactose transport system permease protein | P |  |
| AZ064_02807 | *lacG* | Lactose transport system permease protein | P |  |
| AZ064_03108 | *-* | Response regulator transcription factor | K | - |
| AZ064_03109 | *sasA* | Adaptive-response sensory-kinase | T |  |
| AZ064_03113 | *-* | Hypothetical protein |  |  |
| AZ064_03310 | *dppA (gsiB)* | Bacterial extracellular solute-binding protein | E | - |
| AZ064_03311 | *dppB (gsiC)* | Binding-protein-dependent transport system, inner membrane component | U |  |
| AZ064_03312 | *dppC (gsiD)* | Binding-protein-dependent transport system, inner membrane component | U |  |
| AZ064_03313 | *dppF (btuD)* | Vitamin B12 import, ATP-binding protein | E |  |
| AZ064_03314 | *dppD* | Peptide/nickel transport system, ATP-binding protein | P |  |
| AZ064_03316 | *-* | Hypothetical protein |  |  |

B. Orthogroups found in ≥75% *E. casseliflavus* genomes (n=21) and ≤25% *E. flavescens* genomes (n=10)

| Gene | Putative Gene Name | Annotation | COG | Functional consensus for gene cluster |
| --- | --- | --- | --- | --- |
| AZ179_00010 | *-* | Putative HMP/thiamine permease protein | P |  |
| AZ179_00039 | *lapB* | ATP binding protein for adhesion system | V | - |
| AZ179_00040 | *-* | Transcription regulation | S |  |
| AZ179_00407 | *-* | Hypothetical protein | - |  |
| AZ179_00687 | *-* | Transcription regulation | K | Carbohydrate transport & metabolism |
| AZ179_00689 | *araQ* | L-arabinose transport system permease protein | G |  |
| AZ179_00690 | *lacF* | Lactose transport system permease protein | G |  |
| AZ179_00691 | *-* | Extracellular multiple sugar transport system, substrate-binding protein | G |  |
| AZ179_00692 | *-* | Maltose phosphorylase | G |  |
| AZ179_00693 | *-* | Hypothetical protein | - |  |
| AZ179_00694 | *-* | RDD Family | S |  |
| AZ179_00697 | *-* | Acetyltransferase | K |  |
| AZ179_00698 | *dcuC* | Putative cryptic C4-dicarboxylate transporter | C |  |
| AZ179_00699 | *rihC* | Ribonucleoside hydrolase | F |  |
| AZ179_00700 | *-* | Transcription regulation | K |  |
| AZ179_00936 | *-* | Cof-type HAD-IIB family hydrolase | S |  |
| AZ179_01136 | *clsA* | Major cardiolipin synthase | I |  |
| AZ179_01160 | *-* | Cation active transporter | P | - |
| AZ179_01164 | *uvrA* | DNA repair system protein A | L |  |
| AZ179_01165 | *-* | MarR transcription regulator | K |  |
| AZ179_01227 | *-* | Small inner membrane protein | S | Function Unknown |
| AZ179_01228 | *mepA* | Multidrug export protein | V |  |
| AZ179_01229 | *mepR* | Transcription regulator | K |  |
| AZ179_01230 | *rsiV* | Anti-sigma-V factor | S |  |
| AZ179_01232 | *pgdA* | Peptidoglycan-N-acetylglucosamine deacetylase | G |  |
| AZ179_01238 | *-* | Transcription regulation | K |  |
| AZ179_01239 | *hchA* | Protein/nucleic acid deglycase | S |  |
| AZ179_01240 | *-* | Oxidoreductase, NAD dependent epimerase/dehydratase family | GM |  |
| AZ179_01429 | *-* | Toxin-antitoxin system, Vapbc type, antitoxin component | K |  |
| AZ179_01587 | *-* | Enterotoxin | M | Cell wall/membrane/envelope biogenesis |
| AZ179_01588 | *-* | Transferase involved in polysaccharide synthesis | M |  |
| AZ179_01772 | *-* | Alpha-L-fucosidase | G |  |
| AZ179_01863 | *-* | MerR family transcriptional regulator | K | - |
| AZ179_01864 | *-* | tRNA methyltransferase for translation | Q |  |
| AZ179_01900 | *-* | Trans-aconitate 2-methyltransferase | Q |  |
| AZ179_01951 | *-* | Phosphoribosyl-AMP cyclohydrolase | NU | Cell motility |
| AZ179_01958 | *-* | Hypothetical protein; Cell wall adhesion and biofilm formation | N |  |
| AZ179_02153 | *-* | Transcription regulation | KT | - |
| AZ179_02154 | *-* | Calcium-transporting ATPase | P |  |
| AZ179_02168 | *-* | Virulence cell surface protein | S | - |
| AZ179_02170 | *-* | Transcription Activator MGA | K |  |
| AZ179_02176 | *amiD* | N-acetylmuramoyl-L-alanine amidase | NU |  |
| AZ179_02231 | *mazF* | Toxin-antitoxin system, toxin component | T |  |
| AZ179_02277 | *-* | Transcription regulation | K | - |
| AZ179_02278 | *-* | Aldo-keto reductase | C |  |
| AZ179_02279 | *-* | Zeta toxin | S |  |
| AZ179_02280 | *-* | Hypothetical protein | - |  |
| AZ136_02728 | *traM* | Conjugal transfer protein, quorum-dependent | - |  |
| AZ179_02409 | *-* | Hypothetical protein | - |  |
| AZ179_03149 | *-* | Transcription regulation | K | Transcription |
| AZ179_03150 | *qorB* | NAD(P)H dehydrogenase (quinone) | GM |  |
| AZ179_03151 | *-* | Protein-ADP-ribose hydrolase | S |  |
| AZ179_03152 | *-* | Protein ADP-ribosyltransferase | K |  |
| AZ179_03153 | *-* | Pyridoxamine 5'-phosphate oxidase | C |  |
| AZ179_03154 | *mutT* | Nudix hydrolase 8-oxo-dGTP diphosphatase | F |  |

**Supplemental Datasets** (Separate Excel Files)

**Dataset S1**: Nearest neighbor analysis for novel species

**Dataset S2**: Unique orthogroups from novel species
